# Genomic and Genotoxic Responses to Controlled Weathered-Oil Exposures Confirm and Extend Field Studies on Impacts of the Deepwater Horizon Oil Spill on Native Killifish

**DOI:** 10.1371/journal.pone.0106351

**Published:** 2014-09-10

**Authors:** Whitney Pilcher, Scott Miles, Song Tang, Greg Mayer, Andrew Whitehead

**Affiliations:** 1 Department of Biological Sciences, Louisiana State University, Baton Rouge, Louisiana, United States of America; 2 Department of Environmental Sciences, Louisiana State University, Baton Rouge, Louisiana, United States of America; 3 Department of Environmental Toxicology, Texas Tech University, Lubbock, Texas, United States of America; 4 Department of Environmental Toxicology, University of California Davis, Davis, California, United States of America; Northwest Fisheries Science Center, NOAA Fisheries, United States of America

## Abstract

To understand the ecotoxicological impacts of the Deepwater Horizon oil spill, field studies provide a context for ecological realism but laboratory-based studies offer power for connecting biological effects with specific causes. As a complement to field studies, we characterized genome-wide gene expression responses of Gulf killifish (*Fundulus grandis*) to oil-contaminated waters in controlled laboratory exposures. Transcriptional responses to the highest concentrations of oiled water in the laboratory were predictive of field-observed responses that coincided with the timing and location of major oiling. The transcriptional response to the low concentration (∼10-fold lower than the high concentration) was distinct from the high concentration and was not predictive of major oiling in the field. The high concentration response was characterized by activation of the molecular signaling pathway that facilitates oil metabolism and oil toxicity. The high concentration also induced DNA damage. The low concentration invoked expression of genes that may support a compensatory response, including genes associated with regulation of transcription, cell cycle progression, RNA processing, DNA damage, and apoptosis. We conclude that the gene expression response detected in the field was a robust indicator of exposure to the toxic components of contaminating oil, that animals in the field were exposed to relatively high concentrations that are especially damaging to early life stages, and that such exposures can damage DNA.

## Introduction

The Deepwater Horizon (DWH) explosion initiated the largest marine oil spill in history, releasing over 200 million gallons of South Louisiana crude into the northern Gulf of Mexico (nGOM) [1], [2]. Several field studies to date have investigated the direct impacts of crude oil on native wildlife species and the immediate impacts that followed the disaster [3]–[5]. Field studies are crucial for offering insights about risk to resident species within an ecologically realistic context [6]. A general challenge with observational field studies is sometimes lack of ability to directly link a causal agent to a specific biological response. Furthermore, temporal and spatial variation of ecological factors, such as hypoxia, salinity variation, temperature variation, community interactions, and variable population genetic backgrounds, can complicate interpretation of cause-effect relationships in the field [7]. In contrast, laboratory studies have greater power to determine cause and effect relationships through careful control of experimental, environment, and biological variables. However, laboratory-based studies lack ecological realism for various reasons sometimes including oversimplified exposure scenarios and mismatch between focal species and species at ecological risk [8], [9]. Strategically designed and integrated laboratory and field studies can improve environmental risk assessment, since complimentary data from both the field and the laboratory will strengthen causal relationships while linking important effects to those observed in the field [10], [11].

**Figure 1:**
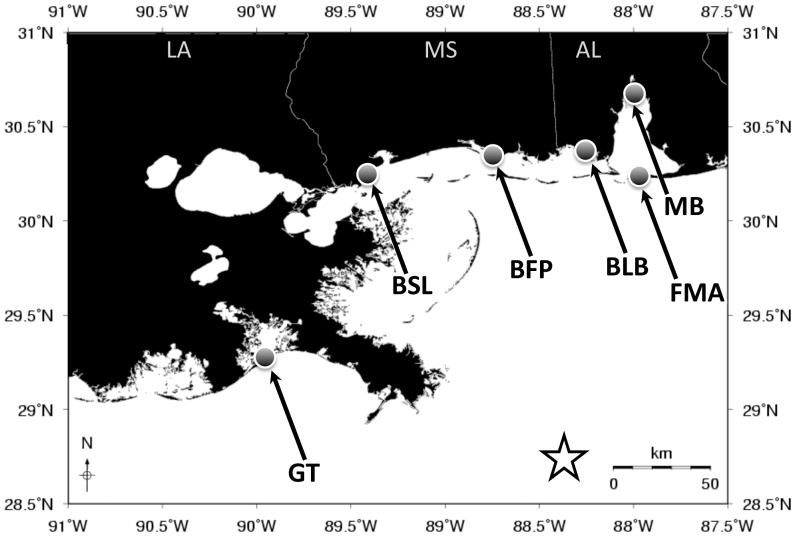
Location of the field study sampling sites from the experiments reported in Whitehead et. al, [20]. Location of field sampling sites, which include Grand Terre (GT), Bay St. Louis (BSL), Belle Fontaine Point (BFP), Bayou La Batre (BLB), Mobile Bay (MB), and Fort Morgan (FMA). The star indicates the DWH spill site.

Gulf killifish (*Fundulus grandis*) are an important model species for estimating ecological impacts of the DWH oil spill because they are the most abundant vertebrate in at-risk nGOM marsh habitats [12], [13]. Furthermore, data from their Atlantic coast-distributed sister species *F. heteroclitus*, which occupies a similar ecological niche, indicates that they are non-migratory and have high site fidelity [14], [15], are important members of the marsh community [16], [17], and are sensitive to organic pollutants relative to other fish species [18]. For these reasons, *F. grandis* were used in a field study to determine the immediate effects of the oil spill on health and physiology [19], [20]. That field experiment was designed within a Before-After Control-Impact (BACI) framework [6], where adult male *F. grandis* were collected pre-oil (sampling trip 1), during oil (sampling trip 2) and post-oil (sampling trip 3), at six sites across the nGOM (Fig. 1). Remote sensing, analytical chemistry and diagnostic biological responses showed the Grand Terre site had direct contact with oil (by sampling trip 2) while the other five sites had no direct contact with contaminating oil. Fish from the Grand Terre site showed divergent genome-wide gene expression through time, in comparison to the other reference sites, and this divergent expression coincided with the timing and location of oil contamination. Gene expression profiles from liver [20] and gill [19] tissues were diagnostic of exposure to the toxic components of oil, and reflected the types of responses, especially in early life stages, that are expected to precede long-term population-level effects.

The controlled laboratory-exposure study reported here seeks to further characterize the biological response of *F. grandis* specifically to weathered oil, and contribute data to further the interpretation of the biological responses observed in the field. Molecular responses in the field study revealed that resident adult animals had been exposed to biologically relevant concentrations of oil. These biological responses were clear even though the analytical chemistry indicated very low concentrations of oil components in tissues and the water column, but concentrations were very high in underlying sediments. Polycyclic aromatic hydrocarbons (PAHs), which are commonly considered important toxic components of oil, are typically quickly metabolized by animals, so tissue concentrations are not a good proxy for estimating prior exposure. The same is true of chemical profiling of water samples, since they represent a brief snapshot in time. We therefore conducted these laboratory studies to test whether controlled high or low concentration exposures were better predictors of responses observed in the field. Using adult killifish as a surrogate, one can then hindcast relative exposure levels and estimate biological impacts for other potentially more sensitive life stages or species that are important for population and community integrity in nGOM marshes.

In the studies presented here, surrogate south Louisiana crude oil was experimentally weathered by mixing with clean brackish water for 30–40 days, after which the water fraction was isolated from overlying oil, to create a water-accommodated fraction (WAF). Fish were exposed to a range of sub-lethal dilutions of WAF. Gill and liver tissues were preserved for gene expression profiling, and blood samples were preserved for DNA strand break assays. Tissue-specific and dose-specific gene expression profiles were characterized, and compared to gene expression profiles in animals collected from the field during the first two years following the DWH disaster.

## Materials and Methods

### Ethics statement

All animal experiments were conducted in accordance with LSU institutional animal care and use protocols approved specifically for these studies (IACUC protocol # 10-066). Fish were collected from the Louisiana Universities Marine Consortium (LUMCON) facility property in Chauvin, LA, with permission. Fish were maintained, and experiments conducted, in an AAALAC accredited facility at LSU. Fish were sacrificed at experiment termination by decapitation.

### Fish collection

Gulf killifish (*Fundulus grandis*) were collected using minnow traps in Chauvin, Louisiana (29.360016 N, 90.625952 W), which was not impacted by DWH oil. Adult males (5–8 cm in length) were collected and transported to Louisiana State University's Aquatics Facility (Baton Rouge, LA). Fish were maintained in a recirculating system at 24°C for three weeks prior to experimentation. Water salinity was kept at 10 ppt (reverse osmosis purified water mixed with Instant Ocean Sea Salt) which matched the salinity detected at field sites [20], and this 10 ppt water was used for all experiments. Water quality was monitored bi-weekly and included ammonia, nitrate and nitrite tests using commercial kits (API brand). Nitrogenous wastes were undetectable during the acclimation period and dissolved oxygen levels were between 5–8 mg/L. Temperatures were kept between 22–24°C and lighting was kept on a 13 hours light, 11 hours dark cycle,

### Range-finding experiment

A pilot study was performed as a guide to determine a sub-lethal dose range for the definitive experimental exposures. Three gallons of south Louisiana crude oil (Plane's Marketing; Lafayette, Louisiana) was added to 30 gallons of clean brackish water in a 400-gallon fiberglass tank. Between June 8^th^ and July 8^th^, 2012, the 400 gallon tank was exposed to ambient outside conditions (full sunlight), mixed twice daily for five minutes each time, always covered with mosquito net, and covered with a canopy during rain (Table S1 in File S1). After 30 days of weathering the water fraction was allowed to settle for two days before being separated from the floating oil (hereafter referred to as the water accommodated fraction: WAF). We performed no further filtering of the WAF. This method for preparing the WAF is different than some more typical standard protocols (e.g., [21]) primarily because we needed to prepare hundreds of gallons of WAF for our exposure experiments, rather than more typical 1-20 liter volumes that are more amenable to standard protocols. Furthermore, since Macondo well oil was exposed to sea surface conditions for many days to weeks before reaching near-shore habitats, we prepared our WAF over a similar duration, in contrast to more standard protocols which typically mix oil and water for 24-48 hours duration [21]. Our WAF preparation method likely resulted in lower total PAHs in our WAF, compared to what could have been achieved using more standard preparation protocols, since our long duration of mixing likely led to significant oil mass loss (though this was not quantified) and mixing energy was likely much less.

The highest sub-lethal dose was determined by exposing animals to four different dilutions of WAF over seven days, including: 1) 100% WAF, 2) 75% WAF (75% WAF+25% clean brackish water), 3) 50% WAF (50% WAF+50% clean brackish water), and 4) 25% WAF (25% WAF+75% clean brackish water). Fifty percent water changes and water quality measurements were performed daily. Only fish from the 25% WAF treatment survived the full seven-day test (Fig. S1 in File S1), so this was designated the highest sub-lethal dose and therefore used as the highest dose for definitive exposure experiments (see below).

### Definitive exposure experiment

Following the pilot study, a second volume of south Louisiana crude oil was obtained (a gift from the Malka Oil Company) and weathered to generate enough WAF for definitive fish exposure experiments. Similar to the pilot experiment, oil and water were mixed at a 1∶10 ratio, where 20 gallons of south Louisiana crude oil was mixed with 200 gallons of clean water in a 400-gallon fiberglass tank. The oil-water mixture was weathered, as described for the pilot project, for 40 days (Table S2 in File S1). Once the WAF was removed from the tank at 40 days, another 200 gallons of clean brackish water was added to simulate a tidal exchange where underlying contaminated waters are replaced with clean waters. For two days this fresh addition of clean brackish water was mixed with the remaining overlying weathered oil and a new WAF was drawn off from the tank (hereafter referred to as the “tidal” treatment).

The definitive fish exposure experiment consisted of three WAF dilutions: a high concentration (25% WAF), a low concentration, (2.5%), a tidal treatment, and a control treatment consisting of clean brackish water. Fish were exposed in 20-gallon glass aquaria. Water changes (50%) and water quality measurements were performed daily. Ammonia, nitrate and nitrite tests were performed and ammonia never increased past 0.50 ppm in any treatment (Table S3 in File S1), while nitrate and nitrite remained undetectable. Fish were sampled after 1 day, 3 days, and 7 days of exposure, at which times liver, gill, and blood tissues were isolated from six replicate adult male fish (six biological replicates) per treatment. Liver was sampled because it is the main organ for xenobiotic metabolism, and gills sampled because they have a large surface area in direct contact with the external aquatic environment. Liver and gill samples were preserved in RNAlater (Ambion, Inc.). Blood samples for DNA strand break analysis were drawn from the caudal vein into a cryovial containing DMSO and RPMI-1640, and stored at -80°C. Fish handling and dissections were in accordance with an institutional animal care and use protocol approved by Louisiana State University (protocol # 10-066). The field study [20] to which we compare data from these laboratory studies included sampling of the same number of biological replicates from three timepoints. In order to have equivalent statistical power to detect gene-specific treatment effects as the field study, we drew replicate fish per treatment from a single tank, since field studies in essence also sampled replicate fish from a single “tank”.

### Analytical chemistry

Definitive exposure experiment water samples from each WAF dose were collected in borosilicate glass amber bottles with Teflon-lined caps. Organics were solvent-extracted from 4 liters of water sample per treatment using DCM, and concentrated down to 1 ml final volume using a nitrogen blow-down system. Analytical chemistry methods (gas chromatography and mass spectrometry) were the same as reported in [20].

### DNA damage

Assessment of DNA damage following exposures was performed on whole blood that was collected by caudal puncture and stored in 10% DMSO, 85% RPMI-1640, and 5% fetal bovine serum. This suspension was frozen overnight at −20°C and then transferred to −80°C until use. Cells from collected whole blood were suspended at 2.5×10^5^ cells per ml in PBS and combined with molten low-melt agarose at a ratio of 1∶10 (v/v). Then 50 µL of the cell-laden agarose was applied to a CometSlide (Trevigen), allowed to solidify at 4°C in the dark for 30 minutes and then lysed by immersion in pre-chilled lysis solution (2.5 M NaCl, 100 mM EDTA, 10 mM Tris, pH 10, 10% sodium lauryl sarcosinate) containing an additional 10% DMSO at 4°C. After 1 hour, the slides were immersed in digestion solution (2.5 M NaCl, 100 mM EDTA, 10 mM Tris, pH 10) with 1 mg/ml proteinase-K (Roche) for 2 hours at 37°C. Slides were washed by immersion in 50 mL pre-chilled 1X neutral electrophoresis buffer for 30 minutes at 4°C. Slides were placed in the CometAssay ES tank containing 1000 mL pre-chilled 1X neutral electrophoresis buffer, and electrophoresis was performed at 20 volts for 10 minutes at 4°C. After electrophoresis, slides were immersed in DNA precipitation solution for 30 minutes, then immersed in 70% ethanol for 30 minutes and air-dried at room temperature overnight. After fixation, slides were stained with 50 µl of SYBR Green I for 1 hour. Slides were viewed utilizing epifluorescence microscopy (Nikon Eclipse Ti-E) and images were analyzed with CometScore software (TriTek Corp.). A total of 100 randomly selected cells (∼50 cells from each of two replicate wells) were scored from each individual. To evaluate relative amounts of DNA damage, we compared tail moments (olive tail moment  =  tail DNA% X length of tail) generated by CometScore from biological replicates in each of our treatments [22]. Differences between the control and the exposure groups were analyzed by two-way ANOVA (df_%WAF_ = 2, df_time_ = 2, df_interaction_ = 4, df_residual_ = 35) for total number of damaged cells and also within each of four damage categories, where main effects were specified as WAF dose and time. The four damage categories were defined according to numerical scores from CometScore olive tail moment (OTM) calculations as follows: <3 = “No Damage”, 3–10 = “Low Level Damage”, 10–25 = “Moderately-Low Level Damage”, 25–50 = “Moderately-High Level Damage”, >50 = “Highly Damaged”. Tukey comparison of means was utilized for post-hoc tests.

### Genome-wide gene expression

Genome-wide gene expression profiling offers a global discovery-based approach for revealing the mechanisms that animals utilize to respond to environmental stressors. Gene expression responses were measured using custom oligonucleotide microarrays (Agilent eArray Design ID 027999). This same microarray design was used in killifish PCB-126 exposure experiments [23] and field-based DWH oil exposure experiments [19], [20], such that data from these studies are directly comparable. The genome response was determined in both livers and gills that were sampled from the control, low concentration (2.5% WAF), high concentration (25% WAF) and tidal treatments. RNA extraction and microarray hybridizations followed the same methods as those reported in Whitehead et al. [20]. Briefly, total RNA was purified using RNeasy spin columns (Qiagen) following tissue homogenization in TRIzol reagent (Invitrogen), followed by preparation of antisense RNA (aRNA) using the amino allyl aRNA amplification kit (Ambion), dye coupling (Alexa Fluor dyes 555 and 647; Molecular Probes), and hybridization to microarrays. Five biological replicates were included per treatment where each sample was hybridized twice including a dye swap. Total RNA and aRNA quality was assayed for quality control using microfluidic electrophoresis (Experion instrument and reagents, Bio-Rad Laboratories, Inc.). Using JMP Genomics (SAS Inc.) data were lowess-normalized, then mixed-model normalized with “dye” and “array” specified as fixed and random effects, respectively, then quantile normalized. The normalized data were then analyzed using mixed model analysis of variance with “dose” and “day” specified as main effects, “dye” was considered a fixed effect, and “array” and within-treatment biological replicates (N = 5) were treated as random effects. Significant treatment effects were determined by setting the p value threshold at <0.01, and false discovery rate was calculated using Q-value [24]. Principal components analysis was used to summarize the trajectories of expression change through time for sets of genes, using MeV software [25]. MeV was also used for clustering of co-expressed genes and generation of heatmaps. Gene ontology enrichment analysis was performed using DAVID Bioinformatics Resources [26], and network analysis performed using Ingenuity Pathway Analysis software (Ingenuity Systems, Inc.). Microarray data have been deposited in EBI ArrayExpress (Accession E-MTAB-2834 for gill data, and E-MTAB-2841 for liver data). Tables S4 and S5 in File S1 lists all genes included in the analyses for gill and liver, respectively, including average expression levels per treatment and p-values from statistical analyses.

## Results and Discussion

The purpose of the range finding experiment was to determine the highest WAF exposure that was non-lethal. Following seven days exposure, the 25% WAF was the highest of the concentrations we tested that caused no mortality (Fig. S1 in File S1). For definitive exposures, no mortalities were observed over the seven-day experiment.

### Analytical chemistry

The four WAF treatments from the definitive exposures were analyzed to determine the composition of the alkane and aromatic components. The low concentration (2.5% WAF) exhibited the fewest alkane and aromatic components above detection limit, and for those above detection limit, the 2.5% WAF treatment had the lowest concentrations compared to 25% and tidal WAFs (Table S6 in File S1). Compared to the 2.5% WAF, the 25% WAF contained approximately 10-fold higher concentration of total alkanes, and approximately 12-fold higher concentration of total aromatics. The tidal treatment was most similar in chemical composition to the high concentration. It appears that once the original WAF was replaced with new clean brackish water (the “tidal flush”), much chemical remained in the overlying oil to partition into the water column. It should be noted that oil is a highly complex mixture of chemicals that is not thoroughly characterized by our selected set of 71 analytes, such that unmeasured components of oil may contribute to the biological responses that we detect.

### DNA damage

**Figure 2:**
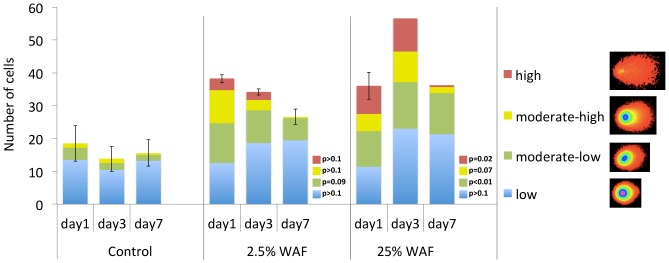
DNA strand breakage (comet assay) after 1 day, 3 days, and 7 days exposure to a control treatment, a 2.5% WAF treatment, and a 25% WAF treatment. Stacked bars represent the average number of cells showing strand breakage within each of four categories of severity (low, moderately-low, moderately-high, and high DNA damage as assessed by tail moment) for each treatment and time point. Comet images are representative of each category of damage. P-values are indicated for results from post-hoc tests that compare treated to control samples across all days within each severity level. Results from post-hoc tests indicate that, relative to control, DNA damage was elevated in the 25% WAF treatment, especially for high levels of damage.

We determined that DNA damage was a direct response to the exposure of the dissolved chemicals in the WAF dilutions that persisted through time and varied by dose compared to control by performing single cell gel electrophoresis on whole blood (Fig. 2). When assessing the total number of damaged cells (OTM>3), the main effect of oil treatment (% WAF) significantly increased tail moments and accounted for 22.9% of the total variance that was observed with respect to control (p = 0.007). However, neither time nor an interaction of time and dose accounted for a significant percent of the variance observed with regard to total number of damaged cells between treated and un-treated controls. Post-hoc tests indicated that DNA strand breakage was significantly elevated compared to control in the 25% WAF treatment only (p<0.05) for the high damage and moderately-low damage categories, with a trend toward elevation of moderately-high damage (p = 0.07).

Controlled exposures to WAFs from diverse crude oils cause DNA damage in diverse species including fish [27], bivalves and urchins [28], and amphipods [29]. Furthermore, several field studies have determined a cause-and-effect relationship between DNA strand breakage and PAH contamination from oil, for example in fish [30], [31] and mussels [32]–[34], and DNA adducts may also be elevated in fish exposed to oil-derived PAHs in the laboratory and field (e.g., [35], [36]). Depression of successful cell division, commonly considered a proxy for genetic toxicity, was elevated in Pacific herring larvae exposed to oil from the Exxon Valdez oil spill (EVOS) in the field and laboratory [30], [37], where genotoxicity was correlated with other effects that are commonly induced by oil including increased morphological deformities and pericardial edema. We conclude that the WAF of south Louisiana crude oil is capable of causing DNA damage in exposed individuals.

### Genome-wide gene expression

**Figure 3:**
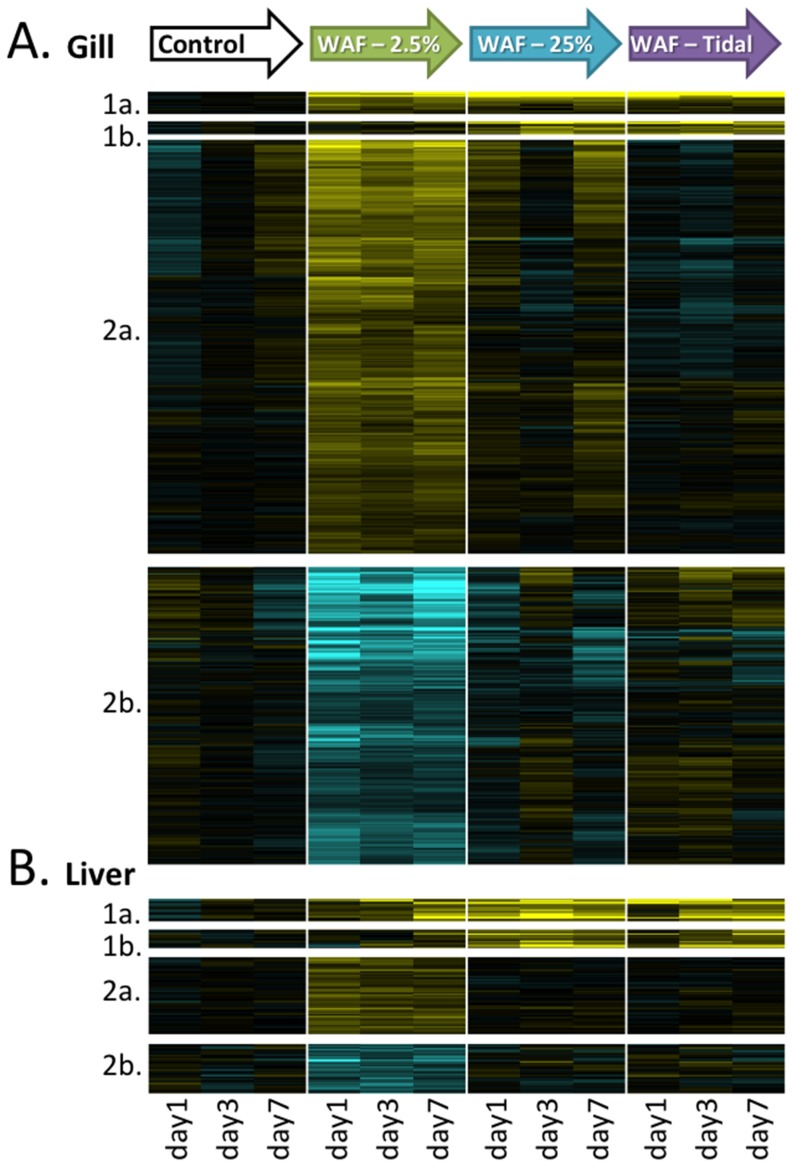
Patterns of expression for significantly WAF-responsive genes across treatments and time points for gill (A) and liver (B). Each row represents a gene, and columns represent consecutive sampling times (1 day, 3 days, 7 days) within a WAF treatment. Cell color indicates an up regulation (yellow) or down-regulation (blue) compared to the control treatment. Genes are grouped by patterns of co-regulation. Four patterns of co-regulation were observed relative to the control for both tissues, including up-regulated for all WAF concentrations (cluster 1a), up-regulated for the high and tidal concentrations only (cluster 1b), up-regulated for the low concentration only (cluster 2a), and down-regulated for the low concentration only (cluster 2b). Scale indicates fold-level of up- or down-regulation.

We observed two main gene expression patterns that were common for both tissues. One cluster of co-expressed genes was up-regulated at all WAF treatments (gill = 17 genes, Fig. 3A, cluster 1a; liver = 17 genes, Fig. 3B, cluster 1a) or at least the high and tidal doses (gill = 10 genes, Fig. 3A, cluster 1b; liver = 14 genes, Fig. 3B, cluster 1b) compared to the control. Another larger set of genes was differentially expressed at the low concentration only relative to the control. The gene expression response was very similar between the high and tidal treatments, consistent with the similar chemical profiles for these two treatments. We hereafter refer to the 25% WAF and tidal treatments as the high concentration treatments.

Gene ontology (GO) enrichment analysis of sets of co-regulated genes can offer insight into the molecular mechanisms that underpin biological responses. The gene sets that were WAF-responsive at the high concentrations or in all three WAF treatments (Fig. 3A cluster 1) were significantly enriched for genes associated with the KEGG pathway “metabolism of xenobiotics” (hsa00980) (p = 0.004). These genes include well-known transcriptional targets of the pollutant-activated AHR signaling pathway (CYP1A1, CYP1B, CYB5, UGT, FOXQ1). This AHR pathway is canonically activated by organic pollutants such as those found in oil (e.g., PAHs). In adult fish, AHR activation enables metabolism of PAHs. As such, activation in adults may not indicate toxicity, though AHR activation at high PAH doses can mediate DNA strand breakage [38]. In embryos, inappropriate AHR activation can cause developmental abnormalities [39], [40], though not all of the developmental toxicity from oil exposure is mediated through this signaling pathway [41]. The activation of the AHR pathway is clear evidence of biologically-relevant exposure to the toxic components of weathered oil that are available in the WAF, even at the lowest dilution (2.5% WAF). Divergent gene expression associated with the timing and location of oil contamination in the field also implicated activation of the AHR signaling pathway [20].

The largest cluster of co-expressed genes was comprised of those that were up- and down-regulated in the low concentration only in both gill and liver (gill = 853 genes, Fig. 3A, cluster 2; liver = 95 genes, Fig. 3B, cluster 2). The gene set that was up-regulated at the low concentration only (Fig. 3A cluster 2a) is enriched for GO terms including *nucleotide binding* (p = 3.0E-6, number of molecules = 90), *positive regulation of transcription* (p = 2.8E-4, n = 22), *centrosome* (p = 6.2E-3, n = 10), and *negative regulation of apoptosis* (p = 0.04, n = 15), whereas down-regulated genes at the low concentration only (Fig. 3 cluster 2b) are enriched for GO terms *RNA processing* and *spliceosome* (p = 1.3E-3 and n = 20, and p = 4.7E-3 and n = 10, respectively), *response to DNA damage stimulus* (p = 0.01, n = 8), and KEGG pathway oxidative phosphorylation (p = 2.1E-3, n = 16).

**Figure 4:**
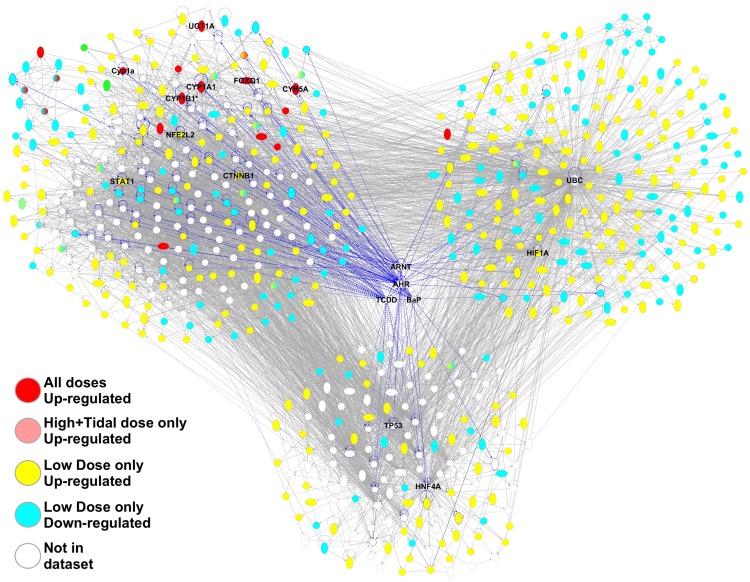
Gill gene interaction network connected by aryl hydrocarbon receptor (AHR) and aryl hydrocarbon receptor nuclear translocator (ARNT) hubs and model toxicants benzo-a-pyrene (BaP) and 2,3,7,8-tetrachlorodibenzo-*p*-dioxin (TCDD). Genes up-regulated at high concentrations (Fig. 2 cluster 1) are colored red, genes that are up- and down-regulated in the low concentration only are colored yellow and blue, respectively. Lines represent interactions between genes, and blue lines highlight genes that directly interact with AHR/ARNT/TCDD/BAP. The same figure, but including names for all genes, is supplied as Figure S2 in File S1.

The genes that were WAF-responsive in gill tissues connect to form three major sub-networks of interacting genes (Fig. 4). Gene functions that are enriched within networks of genes are consistent with the biological functions implicated by GO enrichment analysis. One sub-network (Fig. 4, top left cluster) includes almost all of the genes that were up-regulated at the high concentrations and at all three concentrations (Fig. 3A cluster 1, Fig. 4 red molecules), and is highly connected to AHR/ARNT/TCDD/BaP. AHR and ARNT are key mediators of the toxic response to model toxicants such as TCDD and BaP in diverse species including fish [39], [40]. A second sub-network (Fig. 4, top right cluster) primarily includes genes that were responsive to the low concentration only (Fig. 3A cluster 2, Fig. 4 yellow and blue molecules) and is united by ubiquitin C (UBC). This second sub-network is associated with the functions *processing of RNA* (p = 8.2E-14) and *splicing of RNA* (p = 2.0E-8). The third sub-network, also primarily including genes that were low-concentration responsive only, is associated with functions *cell cycle progression* (p = 7.5E-6) and *tissue morphology* (epithelial cells, p = 2.0E-5).

All three sub-networks are highly connected to AHR/ARNT/TCDD/BaP and to each other. This implies a complex coordinated molecular response to weathered oil, a response that is modular according to exposure concentration, and a response that is functionally coupled to chemicals that are mechanistically related to those that comprise the toxic components of oil (e.g., TCDD and BaP are also AHR agonists). We refer to this molecular response as “modular” since different *sets* of genes are transcriptionally responsive at different doses, rather than a common set of genes with transcript abundance scaled with increasing dose. For example, sub-network 1 includes most of the genes up-regulated by all three concentrations including the high concentrations, which includes many direct transcriptional targets of an activated AHR such as xenobiotic metabolism genes CYP1A1, CYP1B1, CYB5, and UGT, transcription factor NFE2L2, and transcription factor FOXQ1 which when up-regulated by TCDD-activated AHR is associated with developmental abnormalities in zebrafish [42]. Many genes in sub-network 2, which are primarily regulated in the low concentration only, are connected to AHR/ARNT/TCDD/BaP, including the hub UBC which binds both AHR and ARNT [43], [44]. Indeed, UBC is highly connected to genes in sub-network 1 and 3. In addition to UBC, this group of low- concentration-only genes is united by a group of transcriptional regulators, including several known to interact with AHR/TCDD such as STAT1 [45], CTNNB1 [46], [47], NFE2L2 [48], [49], and HIF1A [50]. NFE2L2 may be particularly important, since it is highly connected in our network, it binds with and regulates expression of UBC [51], and is involved in many cell functions including apoptosis, cell death, and cellular response to injury and oxidative stress [52], [53]. Similarly, many genes in sub-network 3 are connected to AHR/ARNT/TCDD/BaP, including the hubs HNF4A which is regulated by ARNT [54], and TP53 which is regulated by AHR/ARNT, TCDD, and BaP [55], [56].

Many more genes were transcriptionally responsive at the low concentration compared to the higher concentrations (Fig. 3). Other studies have detected a similar inverse relationship between the number of differentially expressed genes and chemical dose, including for *Daphnia magna* exposed to cadmium [57], zebrafish exposed to uranium [58], and human lung cells exposed to arsenic [59]. However, in those studies the ratio of the number of low-dose to high-dose genes ranged from 1.1 to 2.5, whereas this ratio was much higher (32) in our study. Clearly, pathways were activated at the low concentration that were not activated by higher concentrations. Given the identity of gene ontologies enriched in this low- concentration-only set (regulation of transcription, centrosome, cell cycle progression, RNA processing, DNA damage, apoptosis), we hypothesize that animals exposed to low concentrations were invoking cellular responses to compensate for exposure to potentially damaging agents in the WAF, and that these compensatory responses were overwhelmed or otherwise inhibited at higher concentrations. Indeed, this is consistent with low toxicity (low DNA damage) in low-concentration fish compared to fish in the control treatment and higher exposure concentrations (Fig. 2).

Compared to gill tissues, fewer genes were transcriptionally-responsive in the liver. Despite these differences, the biological functions that are implicated in response to oil-contaminated water exposures were similar between tissues, especially for high concentrations. For example, genes that were responsive at all three WAF treatments in liver (Fig. 3B cluster 1) were significantly enriched for genes associated with the KEGG pathway “metabolism of xenobiotics” (hsa00980) (p = 0.004). As in gills, these genes include well-known transcriptional targets of the toxicant-activated aryl hydrocarbon receptor (AHR) signaling pathway (e.g., CYP1A1, CYP1B, UGT, GST).

**Figure 5:**
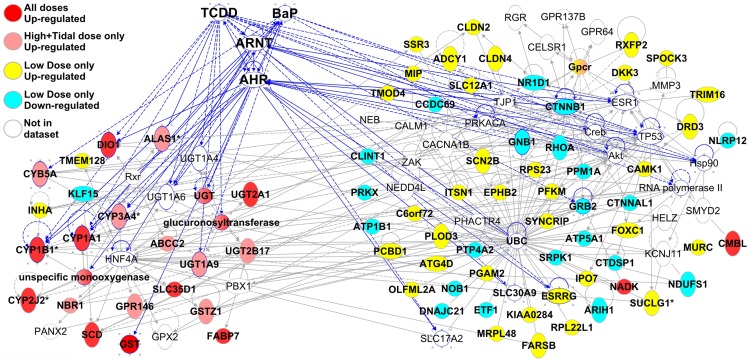
Liver gene interaction network connected by aryl hydrocarbon receptor (AHR) and aryl hydrocarbon receptor nuclear translocator (ARNT) hubs and model toxicants benzo-a-pyrene (BaP) and 2,3,7,8-tetrachlorodibenzo-*p*-dioxin (TCDD). Genes up-regulated at high concentrations (Fig. 2 cluster 1) are colored red, genes that are up- and down-regulated in the low concentration only are colored yellow and blue, respectively. Lines represent interactions between genes, and blue lines highlight genes that directly interact with AHR/ARNT/TCDD/BAP.

In liver, WAF-responsive genes connected to form two coupled sub-networks (Fig. 5). One sub-network primarily includes genes up-regulated at all concentrations and high+tidal concentrations (Fig. 5, left cluster), and largely represents genes activated by ligand-activated AHR, similar to the response in gill. A second network is united by UBC and TP53 hubs (Fig. 5, right cluster). These hubs were also implicated in the gill analysis, but in the liver the genes associated with the UBC hub are not associated with RNA processing functions as they were in the gill network. In fact, within the UBC-centered networks of both liver and gill, only 5 genes are shared between the two tissues (PLOD3, FOXC1, FARSB, ITSN1, and SRPK1).

Genes that were transcriptionally responsive to a high concentration of oil in liver and gill were largely overlapping in gene identity and gene function. In contrast, genes that were low-concentration responsive only were largely non-overlapping in identity or function between tissues. This implies that at high concentrations, the two tissues respond similarly at the molecular level, and this response is largely explained by activation of the AHR signaling pathway, though at low concentrations the two tissues diverged in their molecular response. In gills the low-concentration response appears to be associated with regulation of cell cycle, transcription, DNA damage, and apoptosis, which may be protective from acute toxicity. However, in the liver few functional categories were implicated, perhaps because the internal dose was insufficient to have induced compensatory responses over the duration of this experiment, or the liver was more efficient at clearing damaging chemicals compared to gill. Interestingly, in response to oiling in the field study, more genes were responsive in the liver than in the gill [19]. This phenomenon perhaps reflects differences in the nature of contaminant exposures in the field compared to in the simplified exposure regime of the laboratory. For example, in the laboratory, routes of contaminating oil entry into fish were likely primarily through the gill, since animals were exposed for a relatively short duration, and to only the WAF. This is in contrast to the field, where animals were exposed for much longer than 7 days [20], and presumably exposed to chemicals in the WAF and to chemicals bound to suspended particles which likely gained entry primarily through gills, and exposed to contaminants ingested through their gut (bound to suspended particles and in food). The field-exposure regime may have precipitated a greater internal dose, thereby initiating a stronger biological response in the liver. These hypotheses for tissue-specificity in molecular responses to contaminating oil under different exposure regimes merit further detailed exploration.

**Figure 6:**
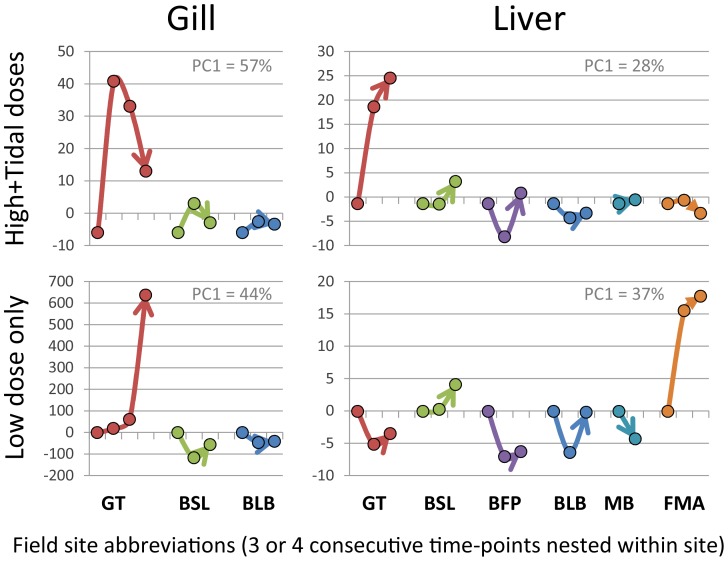
Trajectories for transcriptional change through time and across sites in the field study [20] for subsets of genes identified in the laboratory study reported here. Gill data are illustrated in left column panels, and liver data in right column panels. Principal component 1 (PC1) is plotted following PC analysis, and the proportion (%) of the transcriptional variation across sites and time accounted for by PC1 are indicated in brackets for each plot. Panels arranged by row represent different subsets of genes selected from the laboratory study, including the high-concentration responsive genes (row 1), and all the genes that were differentially expressed in the low concentration only (row 2). In the field study, liver responses were profiled across 6 sites at 3 time points, including Grand Terre Island (GT), Bay St. Louis (BSL), Bayou La Batre (BLB), Belle Fontaine Point (BFP), Mobile Bay (MB) and Fort Morgan Alabama (FMA) [20], before, during, and after (points at base, middle, and tip of arrows, respectively) peak oiling in 2010. The GT site was the only site directly oiled, and oil arrived between the first and second time-points. Gill responses were profiled at the oil-impacted GT site and two reference sites (BSL and BLB), at the same three time-points, plus an additional time-point at the GT site during summer 2011, one year after the third sampling time-point.

Genomic responses from the laboratory study can contribute to interpretation of genomic responses observed in our field studies. In the field, the gene expression response for livers was profiled across six field sites and three time points [20], and the profile for gills was across a subset of field sites (three) and included a fourth time-point for the GT site (one year after the third sampling time) [19]. We selected the sub-set of genes that was transcriptionally-responsive to the high WAF concentrations in each tissue (Fig. 3, cluster 1) and explored the expression of those same genes across sites and sampling times in the field study. Principal components analysis shows that these genes diverged in expression at the GT site at the second sampling time, which coincides with the timing and location of major oiling (PC1 plotted in Fig. 6). For the genes that were transcriptionally responsive to only the low concentration in laboratory studies (Fig. 3, cluster 2), we also explored the spatial and temporal patterns of expression in the field. In contrast to the high-concentration responsive genes, the low-concentration responsive genes did not show divergent expression coincident with major oiling in the field. Interestingly, expression of these low-concentration responsive genes diverged at the fourth sampling time at GT in gills (livers were not profiled at GT time 4) and diverged at the second sampling time at FMA in livers (gills were not profiled at FMA) (Fig. 6). Satellite imagery data [20] and shoreline cleanup assessment technique (SCAT) data (http://gomex.erma.noaa.gov) indicated that undispersed crude oil came close to the FMA field site during second sampling date, and by the fourth sampling date at GT the contaminating oil had been weathering *in situ* for over 1 year. Therefore, patterns of expression of these low-concentration responsive genes in the field are consistent with exposure to low oil concentrations possibly experienced in the field.

We tested whether these patterns of expression in the field study of low and high WAF concentration genes were robust to time-course variation in gene expression. For example, in the gill high WAF concentration there are some genes in cluster 2 (Fig. 3A; low dose responsive genes) that appear to have elevated expression at days 1 and 7 relative to control. We tested for this by excluding day 3 data and re-doing the mixed model analysis. Only 4% of the genes from cluster 2 (Fig. 3A) were significantly differentially regulated in the high WAF concentration relative to control when excluding day 3 data, and none of these genes were also transcriptionally responsive in the tidal treatment. Nevertheless, we re-did the PCA of field data after reassigning this small set of genes from the low WAF responsive set to the high WAF responsive set, and found that this did not alter the trajectories of transcriptome change across field sites and time (Fig. S3 in File S1). We conclude that the predictive value of our data is robust to the subtle time-course variation observed in the lab.

Despite much potential for complicating variation in field studies, the gene expression response that coincided with the timing and location of oiling in nGOM marshes [19], [20] was highly consistent with the gene expression response specifically to weathered oil in the laboratory. High and low concentration exposures caused very distinct gene expression responses in the laboratory; expression of the gene set that was responsive to the high concentration exposure was predictive of the timing and location of major oiling in field-sampled fish, whereas the gene set that was responsive to the low concentration exposure was not. Quantifying oil exposure in the field, especially across space and time, is a difficult task since resident animals are continuously exposed to contaminants in diverse media (water, sediments, suspended particles, prey), and this exposure can vary as animals and food sources move and as environmental conditions change, as oil weathers and degrades in situ, and as new oil arrives.

Since laboratory studies reported here show that the high WAF concentration is highly predictive of the transcriptomic signature from animals exposed to oil in the field, but that the low concentration is not, we can use this to hindcast the types of exposures, at least in a general way, that are likely to have been experienced by the killifish and other organisms in those field sites. Total PAHs (tPAH) summed to ∼300 ppb and ∼3,000 ppb in the low and high-concentration WAFs, respectively. We can therefore predict that, at the time of peak oiling in Barataria Bay, resident organisms were exposed to tPAH concentrations at least between 300 and 3,000 ppb. PAHs were almost non-detectable in the water column during peak oiling at our oil-exposed field sites, but tPAH in shallow marsh sediments ranged from between 284 ppm [19] to over 8,000 ppm [20] in samples collected within three months after peak oiling (note that sediment concentrations reported in Dataset S2 from Whitehead et al. [20] are in mg/kg units). Obviously, these concentrations are not acutely lethal for adult killifish (we were sampling live animals in both field and laboratory studies) though they were genotoxic in our laboratory exposures (Fig. 2). In contrast, early-life stages are much more acutely sensitive to the toxic effects of oil. Sediment tPAH concentrations ∼38 ppm, in sediments contaminated from the DWH disaster, were sufficient to cause decreased heart rates and decreased hatching success in developing killifish [19]. Since hydrocarbons in sediment are less bioavailable that those in the WAF, one would predict early-life toxicity at even lower tPAH concentrations in WAF. Consistent with this, for WAF exposures of pink salmon embryos, tPAH concentrations in the <10 ppb range (between 2–3 orders of magnitude lower than our high-concentration WAF) were sufficient to affect development and impact survival in the lab and field [60], [61]. Though fully developed animals can survive exposures to high concentrations of tPAH, sub-lethal effects, and interactions with other ecological stressors [62], may be expected. Indeed, we show that exposures mimicking those experienced in the field cause DNA strand breakage. Furthermore, tPAH WAF concentrations ∼100 ppb (nearly 2 orders of magnitude lower than our high-concentration WAF) caused osmoregulatory impairment in exposed juvenile herring, and 40 ppb exposures were sufficient to impair swim performance and increase post-exercise mortality in juvenile herring [63]. We conclude that the genome-wide gene expression response detected in the field was an excellent indicator of exposure to the toxic components of oil, that animals in the field were exposed to relatively high concentrations of contaminants, and that such exposures are well-within the ranges that are capable of causing developmental impacts in early life stages and sub-lethal impacts in adult animals including DNA damage.

## Supporting Information

File S1 **Figure S1.** Survivorship of *Fundulus grandis* throughout the 7-day exposure period for four WAF dilutions during the range-finding exposure experiment. **Figure S2.** Gill gene interaction network connected by aryl hydrocarbon receptor (AHR) and aryl hydrocarbon receptor nuclear translocator (ARNT) hubs and model toxicants benzo-a-pyrene (BaP) and 2,3,7,8-tetrachlorodibenzo-*p*-dioxin (TCDD). Genes up-regulated at high concentrations (Fig. 2 cluster 1) are colored red, genes that are up- and down-regulated in the low concentration only are colored yellow and blue, respectively. Lines represent interactions between genes, and blue lines highlight genes that directly interact with AHR/ARNT/TCDD/BAP. The same figure, but excluding names for all genes, is included in the main manuscript as Figure 4. **Figure S3.** Trajectories for transcriptional change through time and across sites in the field study (Whitehead A, Dubansky B, Bodinier C, Garcia T, Miles S, et al., 2012, Genomic and physiological footprint of the Deepwater Horizon oil spill on resident marsh fishes. Proceedings of the National Academy of Sciences 109: 20298-20302.) for subsets of genes identified in the laboratory study reported here. Principal component 1 (PC1) is plotted following PC analysis, and the proportion (%) of the transcriptional variation across sites and time accounted for by PC1 are indicated in brackets for each plot. Panels arranged by row represent different subsets of genes selected from the laboratory study, including the high-concentration responsive genes (row 1) following re-analysis that excludes the 3-day sampling timepoint (see text), and all the genes that were differentially expressed in the low concentration only following re-analysis that excludes the 3-day sampling timepoint (row 2). Gill responses were profiled at the oil-impacted GT site (Grand Terre Island) and two reference sites (Bay St. Louis [BSL] and Bayou La Batre [BLB]), before, during, and after (points at base, middle, and tip of arrows, respectively) peak oiling in 2010, plus an additional time-point at the GT site during summer 2011, one year after the third sampling time-point. The GT site was the only site directly oiled, and oil arrived between the first and second time-points. Tables include environmental conditions during weathering of crude oil for the pilot study (S1), environmental conditions during weathering of crude oil for the definitive exposure experiments (S2), ammonia concentrations throughout the definitive exposure experiment (S3), expression data (gene identities, average expression levels, and p-values from statistical tests) for all genes included in the analysis for gill (S4) and liver (S5), and concentrations of alkanes (mg/L) and aromatics (ug/L) from the control and WAF treatments from the definitive exposure experiment (S6).
(ZIP)
