# Supplementary material for: Genomic and Genotoxic Responses to Controlled Weathered-Oil Exposures Confirm and Extend Field Studies on Impacts of the Deepwater Horizon Oil Spill on Native Killifish
Source: PLoS One. 2014 Sep 10;9(9):e106351. doi: 10.1371/journal.pone.0106351 (PMC4160169; doi:10.1371/journal.pone.0106351)

**Table S3.** Ammonia concentrations throughout the definitive exposure experiment for all doses and time points (ppm).


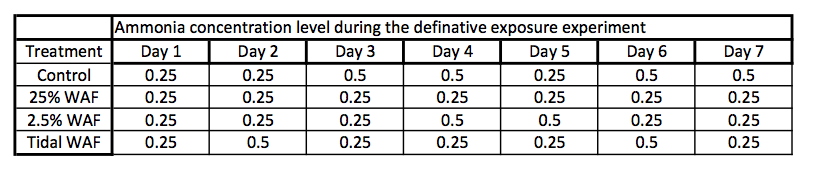

Supplement: File S1 — Figure S1. Survivorship of Fundulus grandis throughout the 7-day exposure period for four WAF dilutions during the range-finding exposure experiment. Figure S2. Gill gene interaction network connected by aryl hydrocarbon receptor (AHR) and aryl hydrocarbon receptor nuclear translocator (ARNT) hubs and model toxicants benzo-a-pyrene (BaP) and 2,3,7,8-tetrachlorodibenzo-p-dioxin (TCDD). Genes up-regulated at high concentrations (Fig. 2 cluster 1) are colored red, genes that are up- and down-regulated in the low concentration only are colored yellow and blue, respectively. Lines represent interactions between genes, and blue lines highlight genes that directly interact with AHR/ARNT/TCDD/BAP. The same figure, but excluding names for all genes, is included in the main manuscript as Figure 4. Figure S3. Trajectories for transcriptional change through time and across sites in the field study (Whitehead A, Dubansky B, Bodinier C, Garcia T, Miles S, et al., 2012, Genomic and physiological footprint of the Deepwater Horizon oil spill on resident marsh fishes. Proceedings of the National Academy of Sciences 109: 20298-20302.) for subsets of genes identified in the laboratory study reported here. Principal component 1 (PC1) is plotted following PC analysis, and the proportion (%) of the transcriptional variation across sites and time accounted for by PC1 are indicated in brackets for each plot. Panels arranged by row represent different subsets of genes selected from the laboratory study, including the high-concentration responsive genes (row 1) following re-analysis that excludes the 3-day sampling timepoint (see text), and all the genes that were differentially expressed in the low concentration only following re-analysis that excludes the 3-day sampling timepoint (row 2). Gill responses were profiled at the oil-impacted GT site (Grand Terre Island) and two reference sites (Bay St. Louis [BSL] and Bayou La Batre [BLB]), before, during, and after (points at base, middle, and [file pone.0106351.s001.zip › TableS3.docx]
